# Community-Based Essential Newborn Care Practices and Associated Factors among Women of Enderta, Tigray, Ethiopia, 2018

**DOI:** 10.1155/2020/2590705

**Published:** 2020-01-20

**Authors:** Gebrehiwot Gebremariam Weldeargeawi, Zenawi Negash, Alemayehu Bayray Kahsay, Yemane Gebremariam, Kidanemaryam Berhe Tekola

**Affiliations:** ^1^Department of Public Health, College of Medicine and Health Science, Adigrat University, Ethiopia; ^2^JSI/Research and Training Institute, Inc., Tigray, Ethiopia; ^3^School of Public Health, Mekelle University, Ethiopia; ^4^School of Public Health, Mekelle University, Tigray, Ethiopia; ^5^Department of Nutrition, School of Public Health, Mekelle University, Ethiopia

## Abstract

*Introduction*. Neonatal mortality contributes a higher percentage of infant mortality, especially in developing countries including Ethiopia where the rate of institutional delivery is low. In Ethiopia, scientific evidences on the level of community-based essential newborn care practice were scanty and inconclusive.

**Objectives:**

The aim of the study was to assess community-based essential newborn care practices and associated factors among women who have infant < 12 months.

**Methods:**

A community-based cross-sectional study was employed among 634 randomly selected women who have infant < 12 months from June 23, 2017, to August 29, 2017, at Enderta district. Data was collected by a face-to-face interview through structured questionnaires, and it was coded, entered, and cleaned using EpiData version 3.1. Then, the data was exported to SPSS version 21 for analysis. Odds ratios and *p* value were computed to know the association between the independent variables with the dependent variable. Finally, a variable at *p* value were computed to know the association between the independent variables with the dependent variable. Finally, a variable at *p* value were computed to know the association between the independent variables with the dependent variable. Finally, a variable at

**Result:**

The overall community-based essential newborn care practice was found 40.7%. Educational status (AOR = 6.6, 95% CI, 2.49-11.97), previous ANC follow-up (AOR = 1.7, 95% CI, 1.2-3.80), weight of the child during birth (AOR = 1.3, 95% CI, 1.12-2.98), and place of delivery (AOR = 2.1, 95% CI, 1.50-4.63) were found to be significantly associated with community-based essential newborn care. Even though overall newborn practice was found to be good, the cord care practices were found to be poor that indicated there is a need to rise community awareness.

## 1. Background

Despite the observed declining in the proportion of deaths among children less than five years, neonatal mortality remains high. Globally, every year, nearly 46% of all deaths in children under five were among newborn infants. According to 2013 report, there were an estimated 2.9 million neonatal deaths annually within the first 4 weeks of their life. Almost 99% of these neonatal deaths occurred in the low- and middle-income countries with the highest rates (38%) in sub-Saharan African accounts globally [1].

Neonatal mortality forms almost two-thirds (1/5 million) of the 8 million annual deaths of children under one and almost 40% of all deaths of children under 5 years [2]. Three major causes, namely, complications of prematurity, intrapartum-related neonatal deaths, and neonatal infections, account for more than 85% of newborn deaths. Complications of prematurity are currently the second leading cause of death of children less than five years [1].

Cognizant of the magnitude of the problem, the World Health Organization has come up evidence-based cost-effective measures to improve neonatal outcomes to be used by all stakeholders who engaged with the neonate care, but the neonatal death still remains very high as compared with the overall child mortality rate [3]. In Ethiopia, the current neonatal mortality was 29 deaths per 1000 live births, which makes the highest of the recent infant mortality rates (IMR) which accounts about 63% [4]. Lack of knowledge on the primary caregiver and coupled with strong cultural beliefs influence neonatal survival because newborn care practices by the primary caregiver immediately after birth are important in determining neonatal mortality and morbidity rate [5].

Study conducted in Gulomekeda, Ethiopia, revealed that about 80.4% of mothers had good knowledge on essential newborn care, but 60% of mothers applied butter or oil on the cord stump [6]. Similarly, Ethiopia is one of the ten countries with the highest number of neonatal deaths globally, with an estimated 122,000 newborn deaths per year [7]. Another report from the country revealed that three quarters of newborns had clean cord cutting and tying of the umbilical cord, but only 12% of the mothers had a postnatal checkup within two days, and the quality of the health services they provided was very poor [8]. To alleviate this problem, the government of Ethiopia put efforts to implement the health policy and commitment to achieve the SDG by focusing mother and child health as its top priority [9]. Accordingly, the Federal Ministry of Health (FMOH) introduced prenatal and postnatal home visits by health extension workers (HEWs) in all parts of the country [9, 10] because majority of the neonatal deaths that occurred were preventable because the death is strongly associated with inadequate health care that the mother receives before and during pregnancy, childbirth, and the postnatal period [11–13] due to unavailability of the services, inadequate number of skilled personnel, geographical inaccessibility and poor quality of care, financial constraints, cultural practices, mothers awareness/knowledge about newborn care, maternal health and sociodemographic characteristics, and educational status of mothers [14, 15]. Thus, to decrease this neonatal morbidity and mortality rate, primary caregivers should have the appropriate knowledge and practices on essential neonatal care, but the available literature in Ethiopia did not provide much information about mothers' knowledge and associated factors on essential newborn care. Thus, in order to generate information on essential newborn care practice, this study has been conducted among those women who have an infant of less than 12 months, and it contributes for a careful design of initiative that was targeted to address this group of population through promoting community-based strategies.

## 2. Methods

### 2.1. Study Area and Design

The study was conducted in Enderta district, Tigray, Ethiopia, from December 2017 to March 2018. The district is found in the southern east of Tigray regional state, North Ethiopia. A community-based cross-sectional study design was employed among 634 women who had infants < 12 months. Women who had infants < 12 months were considered the as source population of the study, and randomly selected women who had infants < 12 months during the data collection period were considered the sample population. All women who had infants < 12 months prior to the data collection period of the study and who live in the study area for at least 6 months were included.

### 2.2. Sample Size and Techniques

The sample size was calculated using a single population proportion formula. According to the Ethiopian Demographic Health Survey 2011, the prevalence of initiated breastfeeding within one hour after delivery (one of the contributing factor) was 52% [4]. Consideration at 95% CI, 5% margin of error, and 10% nonresponse rate, the total sample size was 634 women. A multistage sample technique was employed to recruited participants. Five rural kebeles and two semiurban kebeles were selected by a lottery method first, and then, the total sample size was distributed randomly proportional to the size of the population in each selected kebeles. In Ethiopia, the health extension workers who are providing services for the local communities registered every mother who delivered an infant. These women who had infants < 12 months were selected through systematic random sampling from a list of women in each kebeles' delivered mothers registration book.

### 2.3. Data Collection Tools and Procedures

Data was collected through a face-to-face interview using pretested structured questionnaires. 10 diploma nurses and three degree midwifery professionals were recruited as data collectors and supervisors, respectively. Two-day training on the purpose of how to collect data and related issues was given for data collectors and supervisors by principal investigators before the actual data collection period. Daily basis checkup for complete filling of the questionnaires was also conducted by supervisors and principal investigators, and completed questionnaires were kept, whereas unfilled and partially filled questionnaires were excluded before analysis.

### 2.4. Data Analysis

Data was coded, entered, and cleaned using EpiData version 3.1 and exported to SPSS version 21 for analysis. Descriptive statistics such as frequencies and percent were computed. Bivariate and multivariable logistic regression analyses were employed. A variable that was significant in the bivariate logistic regression analysis (at *p* value < 0.2) was transferred to multivariable logistic regression analysis. Adjusted odds ratio was computed at 95% confidence interval (CI) to determine the effect of the independent variable to the outcome variable. Finally, a variable with *p* value less than 0.05 was declared statistically significant.

### 2.5. Ethical Approval

Ethical clearance was obtained from the institutional review board of the College of Health Sciences Mekelle University. An official letter of cooperation and support letter were obtained from Tigray Regional Health Bureau; a written letter was also obtained from the district health office of Enderta. Finally, written consent was secured from each participant. The participants were told about the aim of the study, and they were also informed that the information they provided will be kept confidential as the data will be used only for the purpose of generating new information before signing the prepared consent. They were also told they have the right to refuse the interview even in the midterm of the interview if they are incontinent.

## 3. Result

### 3.1. Sociodemographic and Economic Characteristics of the Participants

A total of 634 women who had infant less than 12 months were included in the study. About 202 (31.9%) belong to the age group of 25-29 years with a median age of 28 years. Majority, 517 (91.3%), were married, and two hundred fifty-four (40.1%) were also housewives. Regarding their educational status, 255 (40.2%) study subjects were not able to read and write. Majority, 278 (43.8), of the participants were farmer by their occupation with limited (2.4%) which were students (Table 1).

### 3.2. Obstetrics and Maternal Health Service History of the Participants

Study participants were asked their place of birth for the current infant, and out of the total study participants, 571 (91.1%) gave birth at a health institution. Five hundred fifty-seven (87.9%) had made ANC visit at least once for their recent infant, and 574 (90.5%) reported that they prepared necessary materials for the recent birth. Considering the ANC services, 81.5% of the participants have got counseling services on the advantage of ANC for their recent pregnancy, and 67.4% of them were also vaccinated for TT during ANC visits. Regarding on the post natal services 41.5% were not made post natal services for their recent preganancy.

Among the respondents, 515 (81.2%) heard information about immediate newborn care practice, of which 492 (77.6%) heard from health workers, with small number of them (16.7%) heard from their relatives (Table 2).

### 3.3. Community-Based Essential Newborn Care Practice of Mothers

Participants were asked about the correct practices on cord cutting, cord tying, application on the cord stump, breastfeeding initiation, colostrum feeding, and timing of newborn bathing, immunization, and place of delivery. The overall prevalence of community-based essential newborn care of mother in this was found to be about 40.7%.

Five hundred seventy-three (90.4%) of the respondents have used a new blade as an instrument for cord cutting, but 9.6% used an instrument they had used before. Five hundred three (79.3%) replied that nothing was placed on the umbilical cord of their newborn; however, significant proportion of them (20.7%) did apply something on the umbilicus (butter (74.8%), Vaseline or lotion (19.1%), and caw dug (6.1%)) on the umbilical cord of their newborn. Regarding the materials used for the cord cutting of the newborn, 63 (9.9%) of the participants replied they have used previously used materials. Similarly, about 15% of the participants gave the first milk to the newborn after 24 hours of birth. In addition, 473 (74.6%) of the participants of the study washed their newborn before 24 hours which is very dangerous to the newborn and can cause hypothermia (Table 3).

### 3.4. Factors Associated with Community-Based Essential Newborn Care Practices

In this study, ANC visit, place of delivery, and educational level of participants have been found significantly associated variables with “community-based practice of essential newborn care.” Participants who were able to read and write were 6.6 times more likely practiced ENBC than those who were unable to read and write (AOR = 6.6, 95% CI: 2.49-11.97). ANC visit was also statistically significantly associated with community-based ENBC practice. We found that participants who had ANC visits for the recent pregnancy were 1.6 times more likely practiced than those did not have ANC follow-up (AOR = 1.68, 95% CI: 1.19, 3.80). Again, women who gave birth at a health institution were 2.4 times more likely to practice ENBC as compared with those women who delivered at their home (AOR = 2.14, 95% CI: 1.50, 4.63). Similarly, women who born a child with a birth weight of >2.5 kg were 1.3 times more likely practicing ENBC than those who born a child with a birth weight of ≤2.5 kg (AOR = 1.34, 95% CI: 1.12, 2.98) (Table 4).

## 4. Discussion

The present study found that 90.4% of women practice safe cord cutting using a new blade; this was higher than findings from Nepal (70.7%) [16]. The difference might be explained by the awareness about ENBC which was raised through time since the studies were done at different periods. It could be also due to the sociocultural differences between Ethiopia and Nepal. However, the finding was slightly lower than a study done in Tanzania (95%) [17]. The difference might be due to the awareness of the mothers about transmitted diseases which was better in Tanzania, and it may also be due to the information dissemination in Tanzania which was better than Ethiopia through better health coverage.

The timely initiation of breastfeeding observed in this study also was found 72.2%. This finding was similar with a notational report of EDHS, 2016 (73.6%) [9]; however, it was higher than studies from Tigray, Ethiopia (63%), Goba Woreda, Ethiopia (52.4%), and Addis Ababa, Ethiopia (58.2%) [9, 17–19]. The discrepancies on the finding might be due to mothers which may have better awareness about the advantage of early initiation of breastfeeding, and it could also be due to cultural practices, study area, and study period difference among the study participants.

In our study, women who can read and write were about 6.6 times more likely to report having experiences of community-based essential newborn practice than those who were unable to read and write. This finding was supported by a study conducted in Jimma, Ethiopia [20]. This might be related to the fact that educated mother may have better understanding about the ENBC practices, and they had the chance of accessing information from their schools and by reading books related to community-based essential newborn care. Similarly, women who had a history of ANC visit for the recent baby were more likely practiced as compared to their counterparts. This finding was consistent with a study done in Nepal, Tanzania, and Jimma [15, 16, 20]. This could be explained by women who attended ANC which may get information about the components and the importance of newborn care practice from health care providers.

Again, women who gave birth at health facilities for their recent baby were 2 times more likely reported as they were practiced than those who gave birth at their home. This may be evidenced by mothers who gave birth at health facilities which may have the chance for counseling about the danger of inappropriate child care practices, and in fact, they may have better engagement with health professionals while the problems were faced. Similarly, though the Government of Ethiopia had set a strategy, all mothers who gave birth should be visited by the health extension workers immediately after birth; mothers who gave birth at home were found less likely practicing essential newborn care; this might be because these mothers did not get appropriate information about essential newborn care practices.

Our study revealed that mothers who gave birth a newborn with a birth weight of <2.5 kg were more likely practicing essential newborn care than those who delivered <2.5 kg. This might be explained according to the newborn management guideline of Ethiopia; a newborn with a birth weight of <2.5 kg should be managed as inpatient so that the essential newborn care practices will be provided by health professionals at the health facilities not by the parents themselves.

## 5. Conclusion

In this study, the overall CBEN care was better than from most similar studies of the region; however, their delay for the first bathing was very poor. Educational status of the mother, ANC visit, and place of delivery were found to be significantly associated with essential newborn care (ENBC) practices. Hence, there is a need of improving women education; community mobilization on initiation of breastfeeding and the ANC service utilization should be encouraged.

## Figures and Tables

**Table 1:** Sociodemographic characteristic among women who have a child < 12 months in the rural community of Enderta district, southeast zone, Tigray, Ethiopia, 2017.

Variables		Frequency
Age of the mother	15-19	79 (12.5)
	25-29	202 (31.9)
	30-34	131 (20.7)
	35-39	78 (12.3)
	25-29	202 (31.9)

Marital status	Single	24 (3.8)
	Married	579 (91.3)
	Divorce	31 (4.9)

Monthly income	≤300	91 (14.4)
	301-600	140 (22.1)
	601-1000	101 (15.9)
	>1001	302 (47.6)

Educational status	Unable to read and write	255 (40.2)
	Able to read and write	84 (13.2)
	Elementary 1-8	207 (32.6)
	Secondary and above	88 (13.9)

Religion	Orthodox	610 (96.2)
	Muslim	24 (3.8)

Occupation	Governmental employee	29 (4.6)
	Housewife	254 (40.1)
	Farmer	281 (44.2)
	Daily laborer	24 (3.8)
	Merchant	31 (4.9)
	Student	14 (2.4)

**Table 2:** Obstetrics and maternal health service among women who have children < 12 months in the rural community of Enderta district, southeast zone, Tigray, Ethiopia, 2018.

Variable		Frequency (percentage)
No. of pregnancy	0-1	166 (26.2)
	2-4	353 (55.7)
	≥5	115 (18.1)

No. of birth	0-1	174 (27.4)
	2-4	353 (55.7)
	≥5	107 (16.9)

ANC visit	Yes	557 (87.9)
	No	77 (12.1)

ANC assistance	Physician	8 (1.3)
	Health officer	165 (26)
	Nurse/midwife	360 (56.8)
	HEWs	24 (3.8)

Counseling recent ANC	Yes	517 (81.5)
	No	40 (6.3)

TT vaccination	Yes	427 (67.4)
	No	137 (21.6)

Attending monthly pregnant meeting	Yes	304 (47.9)
	No	330 (52.1)

Preparedness for delivery	Yes	574 (90.5)
	No	60 (9.5)

Materials prepared for delivery	Food	528 (83.3)
	Cloth	544 (85.8)
	Money	384 (60.6)
	Transport	183 (28.9)

Planned birth place	Yes	567 (89.4)
	No	67 (10.6)

Place of delivery	Health institution	571 (91.1)
	Home	63 (9.9)

Delivery attendant	Health profession	571 (90.1)
	TBA	32 (5)
	Relative and friend	31 (4.9)

Mode of delivery	Spontaneous vaginal delivery	544 (85.8)
	Instrumental delivery	52 (8.2)
	C/S	38 (6)

PNC visit	Yes	371 (58.5)
	No	263 (41.5)

Heard about immediate newborn care	Yes	515 (81.2)
	No	119 (18.8)

Source of information	HEW	387 (61)
	Health profession	492 (77.6)
	Mass media	265 (41.8)

**Table 3:** Practice of women who have children less than 12 months on essential newborn care, at Enderta district, southeast Tigray, North Ethiopia, 2017.

Variables	Characteristics	Frequency (percentage)
Essential newborn care practice	Yes	258 (40.7)
	No	376 (59.3)

Where do you put immediately after birth	On the mothers chest	429 (67.7)
	Others	205 (32.3)

Cord cutting instrument	New or boiled razor blade	573 (90.4)
	Used razor blade	61 (9.6)

Material used to tie cord	New string	542 (85.5)
	Other	92 (14.5)

Time of initiation of breastfeeding	Within 1 hour	458 (72.2)
	After 1 hour	176 (27.8)

Frequency of breastfeeding practices per day	<8 times	69 (10.9)
	≥8 times	565 (89.1)

Time of wash for the first time for newborn	After 24 hours	473 (74.6)
	Within 24 hours	161 (25.4)

Time of drying and wrapping the newborn	After delivery of placenta	274 (43.2)
	Do not know	23 (3.6)
	Before delivery of placenta	337 (53.2)

Did your baby received eye ointment from health care provider immediately after birth	Yes	571 (90.1)
	No	63 (9.9)

Did they apply anything on the baby's eye except ordered medication	Yes	267 (42.1)
	No	367 (57.9)

Types of some thing applied on the eye's of new born except ordered medication by health provider	Kuheli	236 (88.3)
	Butter	31 (12.7)

Did you apply anything to the cord	Yes	131 (20.7)
	No	503 (79.3)

What did you do the first milk or colostrum	Gave to baby	603 (95.1)
	Withdraw	31(4.9)

Was your baby vaccinated immediately after birth for BCG on the right	Yes	398 (62.8)
	No	236 (37.2)

What material was used to cut the umbilical cord	New and boiled razor	571 (90.1)
	Previously used razor	63 (9.9)

What material was applied on the cord	Butter applied	98 (73.8)
	Vaseline	25 (19.1)
	Animal dug	8 (6.1%)

Placement of the baby	On the mother's chest/belly	429 (67.7)
	Beside the mother	151 (23.8)
	On newborn bed/table	46 (7.3)
	Do not know	8 (1.3)

Initiation of breastfeeding	Within one hour	518 (81.7)
	2-24 hours after birth	16 (2.5)
	After 24 hours	100 (15.8)

Do you know danger sign	Yes	563 (88.8)
	No	71 (11.2)

**Table 4:** Factors associated with the community-based essential newborn care practices of women who have children < 12 months at Enderta district, southeast Tigray, North Ethiopia, 2017.

Variables	Category	ENBC practice		COR (95% CI)	AOR (95% CI)
		Good practice	Poor practice		
Educational status	Unable to read and write	116	139	1	1
	Able to read and write	22	62	2.352 (1.363, 4.057)^∗^	6.695 (2.494, 11.973)^∗^
	Elementary 1-8	84	123	1.222 (.843, 1.771)	.348 (.179, .678)
	Secondary and above	36	52	1.205 (.738, 1.970)	.746 (.264, 2.107)

Gravidity	1 time	66	100	.559 (.334, .937)	.589 (.220, .978)
	2-4 times	161	192	.440 (.277, .699)	.686 (.314, 1.503)
	≥5 times	31	84	1	1

ANC visit	Yes	238	321	1.838 (1.090, 3.098)	**1.68 (1.19, 3.80)** ^∗^
	No	22	55	1	1

Place of delivery	Health institution	204	290	2.612 (1.529, 4.463)	**2.14 (1.50, 4.63)** ^∗^
	Home	39	24	1	1

Wt. of child during delivery	<2.5 kg	30	68	1.678 (1.057, 2.664)	1.34 (1.62, 2.98)
	≥2.5	228	308	1	1

## Data Availability

The data used to support the findings of this study are available from the corresponding author upon request.

## Abbreviations

ANC: Antinatal care
AOR: Adjusted odds ratio
BSC: Bachelor of science
COR: Crud odd ratio
CI: Confidence interval
EDHS: Ethiopian Demographic Health Survey
ENBC: Essential newborn care
ENCP: Essential newborn care practice
FMOH: Federal Ministry of Health
HEWs: Health extension workers
MPH: Master of public health.

## Additional Points

*Limitation of the Study*. As far as our study was done among mothers who had less than 12 months old children and cross-sectional design, it might be affected by recalled bias.
